# Balancing prior knowledge and sensory data in a predictive coding model of coherent motion detection

**DOI:** 10.1371/journal.pcbi.1013116

**Published:** 2025-05-21

**Authors:** Elnaz Nemati, David B. Grayden, Anthony N. Burkitt, Parvin Zarei Eskikand

**Affiliations:** 1 Department of Biomedical Engineering, The University of Melbourne, Melbourne, Victoria, Australia; 2 Graeme Clark Institute for Biomedical Engineering, The University of Melbourne, Melbourne, Victoria, Australia; Universitatsklinikum Hamburg-Eppendorf, GERMANY

## Abstract

This study introduces a neurobiologically inspired computational model based on the predictive coding algorithm, providing insights into coherent motion detection processes. The model is designed to reflect key principles observed in the visual system, particularly MT neurons and their surround suppression mechanisms, which play a critical role in detecting global motion. By integrating these principles, the model simulates how motion structures are decomposed into individual and shared sources, mirroring the brain’s strategy for extracting coherent motion patterns. The results obtained from random dot stimuli underscore the delicate balance between sensory data and prior knowledge in motion detection. Model testing across varying noise levels reveals that, as noise increases, the model takes longer to stabilize its motion estimates, consistent with psychophysical experiments showing that response duration (e.g., reaction time or decision-making time) also increases under higher noise conditions. The model suggests that an excessive emphasis on prior knowledge prolongs the stabilization time for motion detection, whereas an optimal integration of prior expectations enhances detection accuracy and efficiency by preventing excessive disturbances due to noise. These findings contribute to potential explanations for motion detection deficiencies observed in schizophrenia.

## Author summary

Our study introduces a biologically inspired computational neural model that simulates how the brain balances sensory information and prior experiences to detect motion. The model combines predictive coding, which is a framework where the brain anticipates sensory inputs using past experiences, with a mechanism called surround suppression, which refines motion estimates by comparing central and surrounding visual inputs. Using classic motion perception experiments, the model successfully replicates how humans perceive shared and individual motion in complex visual scenes. Importantly, the model reveals how disruptions in this balance, such as an over-reliance on prior knowledge, may contribute to motion detection deficits observed in individuals with schizophrenia. The findings provide insights into the neural mechanisms underlying motion perception and propose a potential pathway for developing clinical tools to assess and treat visual impairments associated with schizophrenia. By bridging neuroscience, computational modeling, and clinical research, this work highlights the intricate interplay between past experiences and present sensory data in shaping our perception of the world.

## 1. Introduction

Visual motion is a rich and dynamic source of information that allows us to understand our surroundings, track moving objects, and navigate through space. However, motion perception is inherently challenging because the brain must distinguish between self-motion and the motion of objects in the external world. A key aspect of this process is the ability to detect coherent motion, where multiple elements in a scene move together in a structured manner—such as a flock of birds flying in formation or a school of fish swimming collectively. Extracting these global motion patterns from complex visual inputs requires efficient neural mechanisms that integrate local sensory signals while suppressing irrelevant noise. Animal models have provided key insights into the computational strategies used by the early visual system to extract motion cues efficiently. In particular, Gollisch and Meister [1] highlighted how neural circuits in early visual areas process motion by integrating sensory information with internal computations, revealing fundamental principles of motion encoding.

Motion detection is a complex perceptual process that integrates prior knowledge from past visual experiences with current sensory inputs. This process involves neural mechanisms across multiple levels of the visual pathway, particularly in areas responsible for motion perception such as the primary visual cortex (V1 and V2) and the middle temporal (MT) area [2,3]. The activity of the neurons in the visual pathway is strongly influenced by the motion in the surround of their receptive fields [4–6]. Nearby stimuli shape the detection and comprehension of coherent motion [2,3,6]. These mechanisms enable the visual system to integrate motion cues across space and time, shaping motion perception and guiding adaptive behaviors. The microcircuits associated with this process play a fundamental role in integrating motion signals across hierarchical levels, allowing for the distinction between individual and global motion patterns [2,3,6].

### 1.1. Exploring Hierarchical Motion Perception: From Decomposition Principles to Predictive Coding Models

**Fig 1:**
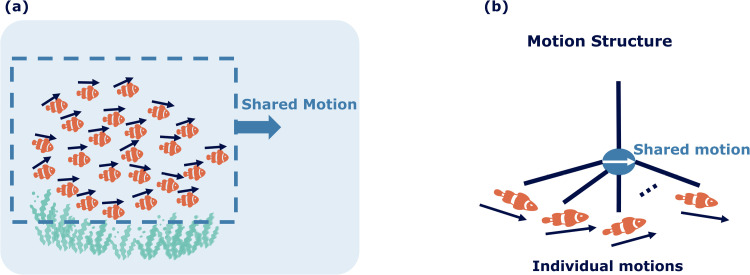
Illustration of visual motion structure separation in complex scenes. (a) A scene of a school of fish, all moving collectively to the right (indicated by the pale blue arrow), with each fish exhibiting its unique motion (indicated by a navy blue arrow above each fish). (b) The scene’s motion can be decomposed into shared motion (averaged over all fish) and individual motion components, relative to the shared motion.

The brain achieves motion perception by using statistical relationships in the velocities of objects [7] and by breaking down object motions into underlying motion sources. These motion sources may not have a one-to-one relationship with objects. For example, for a school of fish, the movement of the fish can be broken down into both the shared motion of the school and the individual motion of each fish, as illustrated in Fig 1. Although it may seem abstract in concept, shared motion significantly enhances our perception and understanding of a visual scene involving many components that display coherent motion. By identifying the underlying motion structure, interpretation of the scene is enhanced, facilitating navigation and prediction tasks.

Pioneering studies by [8] significantly advanced the exploration of hierarchical motion perception by demonstrating the decomposition of motion through displays of moving dots. These studies uncovered that the simple motion of individual components in a visual scene can evoke complex perceptual experiences. This work supports the concept that the visual system uses vector analysis within a scene, distinguishing between the shared motion of objects and their individual motions. In subsequent studies on biological motion, Johansson [9] demonstrated that subtracting the shared motion of components from the visual input enables observers to perceive the relative motions of individual parts. This iterative subtraction method enabled a deeper understanding of the multilevel motion structure in visual scenes, laying the groundwork for our current understanding of hierarchical motion perception.

The vector analysis theory in hierarchical motion perception breaks down object motion into common and relative components, offering insightful yet computationally limited perspectives. While the theory assumes the presence of known motion components and structures, it does not inherently detect these elements from sensory data, thus limiting its application in real-world motion analysis [10]. Complex scenes with multiple possible vector analyses amplify this issue. Researchers have developed several theories to clarify ambiguities in motion interpretation. The ‘minimum principle’, Restle [11] favors the most straightforward interpretations, aligning with Bayesian methods, applicable mainly in ideal, noise-free conditions with specific parametric motions. In contrast, the ‘adjacency principle’ [12] centers on analyzing cues from relative motion between closely located points. Additionally, the ‘belongingness principle’, DiVita and Rock [13] highlights the effects of co-planarity and reference frames on objects in motion perception.

Recently, researchers using normative models based on Bayesian inference over tree structures have gained insights into more intricate aspects of human motion structure perception.[14] introduced a Bayesian framework that uses probabilistic inference over hierarchical motion structures, known as ‘motion trees’, to understand how humans perceive motion structures. Subsequently, Yang et al. [15] proposed that normative Bayesian models over tree structures could explain crucial aspects of motion perception. These studies primarily focused on modeling motion integration in relation to perceptual outcomes and did not address the complex challenge of real-time motion parsing while concurrently inferring scene structure. Bill et al. [7] proposed an innovative approach by formulating visual motion perception as online hierarchical inference within a generative model. This continuous-time model effectively explains various aspects of human motion perception and sheds light on the complex interplay between motion parsing and structure inference in real-time perception.

Motion detection heavily relies on past motion information. Traditionally, researchers have overlooked the role of past information cues and prior experience in the study of motion perception, presenting a significant gap in the understanding of motion processing. Hierarchical predictive coding offers a promising approach to bridge this gap. This framework posits that the brain constructs a multi-level model, where high-level information influences the processing of sensory data at lower levels [16,17]. In motion perception, the brain integrates current visual stimuli with past experiences to predict and interpret motion patterns. The brain efficiently and rapidly processes motion by employing predictive coding at lower levels, such as the primary visual cortex (V1) and V2. This method involves the brain comparing top-down predictions from higher-order areas with bottom-up sensory inputs from these lower-order areas, thereby enhancing the understanding of complex motion structures. Predictive coding actively minimizes errors between expected and actual sensory inputs through feedback mechanisms, refining motion perception and integrating it with more complex functions in higher visual areas like the middle temporal (MT) and inferotemporal (IT) cortices. This active processing outlines the neural structures involved and the underlying processes. Despite the theoretical appeal and potential applicability of predictive coding in the domain of motion perception, the literature still shows a scarcity of such models, indicating a valuable direction for future research.

Motion integration at the biophysical level is thought to occur predominantly in the middle temporal (MT) area of the visual cortex, where neurons are direction-selective and modulate their activity based on the interaction between motion signals in their receptive field and the surrounding context [2,18]. Specifically, MT neurons are known to exhibit center-surround interactions, where the activity is suppressed or enhanced depending on the alignment of motion in the center and surrounding areas [6,19]. The enhancement of neuronal activity facilitates the integration of motion signals, enabling the detection of coherent motion patterns. Our model draws from this theory by incorporating a surround suppression mechanism inspired by these neurobiological processes, allowing it to simulate the contextual modulation of motion signals.

This study aims to explore the brain’s adaptive mechanisms in motion perception through neural connectivity and networks. The central hypothesis of this research is grounded in the concept of ‘predictive coding’ [16], where the activity in the higher levels of the hierarchy—such as the medial temporal (MT) area, which represents a higher order within the visual hierarchy relative to the primary visual cortex (V1)—determines (or ‘predicts’) the activity in the lower levels. Although classical predictive coding functions in this hierarchical fashion, it has also been postulated to be utilized by the brain in the temporal domain to forecast future events [20]. We test the classical predictive coding model with different stimuli and demonstrate that the model successfully extracts the global motion of the stimuli that aligns with perception observations recorded in psychophysical experiments [9,21].

### 1.2. Motion detection deficiency in schizophrenia

Impairments in motion detection have been reported in individuals diagnosed with schizophrenia. Studies have shown that patients exhibit deficits in tasks such as velocity discrimination, where they are required to determine which stimulus is moving faster [22,23]. In an fMRI study, it was observed that the Middle Temporal (MT) area in the visual cortex, which is known to be highly specialized for motion detection, is significantly less active in patients with schizophrenia compared to control subjects when conducting motion detection tasks for direction discrimination and speed discrimination [24]. It is important to note that motion perception does not occur exclusively in the MT area but involves a network of cortical and subcortical areas. Similarly, motion detection impairments in schizophrenia may arise from disruptions across multiple neural structures, not solely the MT area.

A frequently used type of stimulus to investigate the neural circuitry of motion detection is random dots. In the context of schizophrenia, Random Dot Kinematograms (RDKs) prove to be very informative, as they capture the ability of the nervous system to integrate the local motion of individual dots to estimate global motion under different levels of noise. A psychophysical study showed that individuals with schizophrenia require a higher level of coherence in random dots (i.e., less noise) to estimate the global motion of the dots compared to healthy controls [23]. Furthermore, researchers observed that patients with schizophrenia show weaker surround suppression in neuronal circuitry for motion detection than their healthy counterparts [25].

The proposed model, while focused on MT neurons, inspires further investigation into the underlying neuronal circuitry contributing to motion detection deficiencies observed in schizophrenia, including weaker surround suppression and prolonged response times in detecting coherent motion. We aim to highlight that alterations in motion perception, particularly those influenced by surround suppression and prior knowledge weighting, may provide insights into the motion detection impairments commonly observed in schizophrenia. These mechanisms form the basis for explaining why individuals with schizophrenia exhibit prolonged latencies in detecting coherent motion under certain conditions, as demonstrated in psychophysical experiments.

The proposed model is inspired by principles of motion perception observed in the visual cortex, particularly those associated with hierarchical processing and contextual modulation. Rather than explicitly replicating biological circuits, the model incorporates functional properties observed in motion-sensitive cortical areas, such as surround suppression in MT neurons and hierarchical inference mechanisms consistent with the predictive coding framework [6,16].

While the model simplifies these interactions, the chosen areas are supported by neurobiological evidence of their role in motion processing [2,18]. Specifically, V1 and V2 provide the foundational representation of motion signals, which are further integrated and refined in MT and IT through hierarchical predictive coding frameworks [16].

The role of surround suppression in modulating neuronal responses to motion is well established in MT [6,19], and our model incorporates this mechanism as a key component in determining coherent motion perception. This allows our model to replicate psychophysical observations, such as longer response times with increased noise levels.

This study is the first to investigate the necessity of balancing sensory input and prior knowledge in detecting the global motion of stimuli. Despite recent advancements in predictive coding models, no existing model specifically addresses motion detection deficiencies in schizophrenia within the framework of predictive coding. While previous studies have connected predictive coding impairments to broader aspects of schizophrenia such as cognitive and perceptual disruptions [26], our study inspires further exploration into how these impairments specifically affect motion detection.

## 2. Materials and methods

### 2.1. Conceptual overview of the model

The proposed model is built on the predictive coding framework, which is a hierarchical processing mechanism where higher-level areas in the brain generate predictions about incoming sensory data, and lower-level areas compare these predictions with actual sensory inputs to minimize errors. This framework is combined with a surround suppression mechanism inspired by the neurobiological processes observed in the middle temporal (MT) area of the visual cortex. The model incorporates the following concepts:

1. **Predictive Coding:** Sensory inputs are processed through a hierarchy of cortical layers, where each layer computes predictions and sends them to lower layers. Errors between predictions and actual inputs (prediction errors) are propagated upward to adjust the predictions iteratively. This allows the model to dynamically refine its understanding of motion patterns in noisy environments.
2. **Surround Suppression:** A biologically inspired mechanism modulates the activity of motion-sensitive neurons based on the consistency between the motion signals at the center of their receptive field and the surrounding context. When center and surround motions align, the mechanism enhances activity to integrate motion signals. Conversely, when the motions diverge, activity is suppressed to segregate conflicting signals.
3. **Motion Integration:** The model combines the mechanisms of predictive coding and surround suppression to detect global (coherent) motion and filter out irrelevant or noisy motion signals. This interplay between predictive coding and surround suppression allows the model to simulate how the brain processes complex motion patterns.

### 2.2. Model framework

To formalize the process of estimating motion sources for an object, we consider motion dynamics where a set of M hidden motion sources influence the velocity of an object, denoted as $v_{i}$. We represent each of these sources as $r_{j}$ for the $j^{th}$ source. The weight, $u_{i,j}$, quantifies the extent to which each motion source, j, affects the velocity of the object, i. Specifically, we can express the velocity of an object as the sum of the products of the weights and the corresponding motion source contributions,


$${\hat{v}}_{i}=\sum_{j=1}^{M}u_{i,j}r_{j},$$

where ${\hat{v}}_{i}$ represents the estimated velocity of the object i. The weights, $u_{i,j}$, are organized into the matrix U and the motion sources, $r_{j}$, are components of the vector r, which contains the values for all motion sources. The weights, $u_{i,j}$, range from 0 to 1, where 0 indicates no impact and higher values indicate a stronger influence on the object’s motion. It is important to note that the value of each motion source, $r_{j}$, remains consistent across all objects; only the weight, $u_{i,j}$, varies to reflect the total velocity contribution from each motion source to the object. This framework provides a structured approach for dissecting and analyzing the complex interactions between objects and their motion sources, thereby facilitating a deeper understanding of motion dynamics.

We present a methodology to explain the complexities of motion sources and their structural underpinnings. This approach builds on the classical predictive coding framework [16] and incorporates a surround suppression mechanism. This enhancement improves the representation of the interplay between historical motion data and the influence of neighboring objects. The predictive coding framework estimates motion by analyzing neuronal activity within receptive fields and combining it with synaptic weights. Neuronal activity identifies the motion’s source, while synaptic weights quantify how strongly a motion source influences an object’s overall motion. Neurons within receptive fields determine the perceived motion direction, while synaptic weights capture the contribution of each object’s motion to the observed scene dynamics.

**Fig 2:**
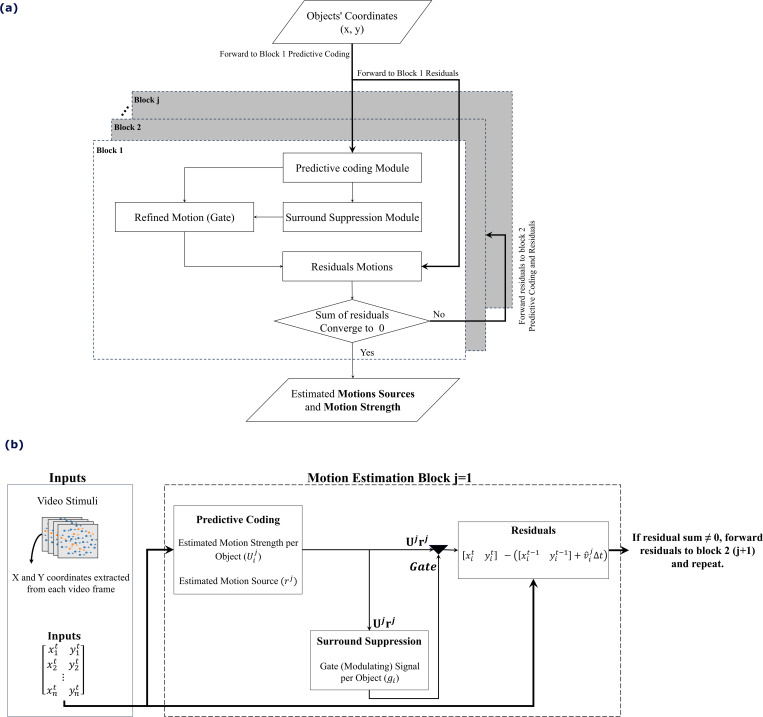
Proposed Motion Estimation Method. (a) Flowchart of the iterative process involving predictive coding, surround suppression, and residual checks. (b) Block diagram showing how video-derived object coordinates are used to estimate motion sources and strengths, with surround suppression gating these estimates based on local consistency. Residual errors drive additional iterative refinements until they converge to zero.

We extend the predictive coding model by incorporating surround suppression, a neurobiological mechanism that adjusts neural excitability based on motion context from adjacent stimuli [27]. Surround suppression fine-tunes the initial motion predictions that predictive coding generates. Fig 2b uses a triangle symbol to represent this refinement process. Predictive coding calculates initial estimates as the product of neuronal activities and synaptic weights, Ur. Surround suppression evaluates the consistency of motion signals and gates these estimates based on the alignment between center and surround motion.

Fig 2a shows how the proposed model processes object positions from video frames. These inputs go to the predictive coding module, which estimates each object’s motion source and strength. The surround suppression module then checks how well each object’s motion aligns with its local context, reducing activity when inconsistencies appear. Next, object positions are updated based on the refined estimates, and residual errors are computed by comparing predicted and actual locations. If these errors converge to zero, the estimates are considered accurate; otherwise, the residuals are fed back for further refinement.

Fig 2b details each step explicitly. The predictive coding module calculates motion strengths and sources for each object as neural activities r weighted by U, yielding estimated velocities $\hat{v_{i}}=U_{i}^{j}r^{j}$, where j indexes the motion estimation block and i indexes the object. These estimates are then gated by the surround suppression module (represented by triangles), which adjusts them according to nearby motions. To update each object’s position, the gated velocity estimate is added to its previous location. If residual errors persist, the process iteratively repeats, incrementally refining predictions until convergence and accurately describing the observed motions.

We systematically examine two-dimensional spatial configurations of objects in visual stimuli using a predictive coding model enhanced with a surround suppression module. This model identifies and quantifies motion sources while considering the influence of adjacent objects. Surround suppression activates when an object’s motion deviates significantly from its surroundings and reduces the impact of these outliers on the motion estimation process. When center and surround motions align, surround suppression remains inactive, allowing accurate motion predictions. This process aligns with findings from Eskikand et al. [28], showing that suppressive mechanisms enhance motion estimation by adapting neural responses to contextual motion cues.

Incorporating this approach, we are able to extract both the global shared motion and individual motion sources, thereby providing a detailed dissection of motion perception. This methodology, which marries the classical framework of predictive coding [16] with a surround suppression mechanism, not only enhances the model’s ability to capture the nuanced interplay between historical motion data and the effects of proximal stimuli but also enables the differentiation between shared and unique motion dynamics, as elaborated in subsequent sections.

### 2.3. Classical Predictive Coding

**Fig 3:**
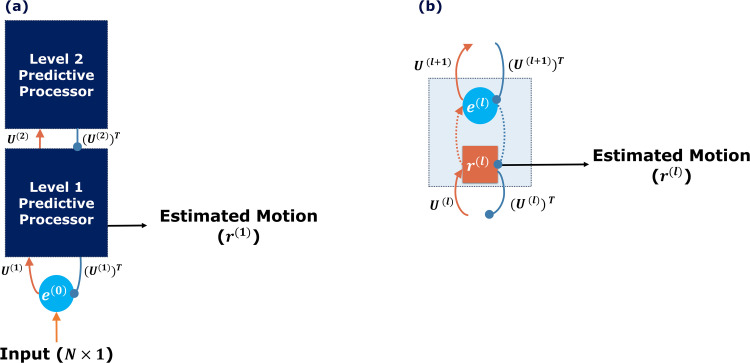
Classical Predictive Coding for motion processing. (a) Two-level classical predictive coding. (b) One level of a predictive processor. Both subplots illustrate the network’s outputs with black arrows. Excitatory and inhibitory connections are highlighted in orange and blue lines, respectively. Error units are depicted as blue circles, while activity units are shown as orange rectangles. Synaptic weights, marked with $U^{\left(l\right)}$, are displayed on the arrows, where l indicates the layer in the hierarchy. Dotted orange and blue arrows represent internal excitatory and inhibitory connections. Here, l is the level index, and N is the number of input objects [16].

The predictive coding model offers a compelling framework for understanding brain function, likening it to an algorithm that predicts future events based on sensory data. The brain continuously makes educated guesses about incoming sensory information and adjusts these predictions as new data arrives. This model organizes its processes in a multilevel structure, as shown in Fig 3a, with each level playing a unique role in processing information.

The predictive coding model centers on two types of units within each level: error units and activity units, represented by the blue circle and the orange rectangle in Fig 3b, respectively. Error units identify mismatches in the brain by comparing predictions against sensory inputs to pinpoint discrepancies, termed “prediction errors”, and relay these errors to subsequent levels in the hierarchy. Activity units create predictions from sensory data and previous prediction errors, transmitting these predictions to the preceding level in the hierarchy.

The predictive coding model aims to minimize the prediction error at each level of the hierarchy within the network. The model achieves this by implementing gradient descent on the prediction errors to adjust connection strengths within the network accordingly. The objective function, E, represents a sum-of-squares error,


$$E=\frac{1}{2}\sum_{l}{\left(e^{\left(l\right)}\right)}^{2}=\frac{1}{2}\sum_{l}{\left(r^{\left(l-1\right)}-U^{\left(l\right)}r^{\left(l\right)}\right)}^{2},$$

where $e^{\left(l\right)}$ represents the prediction error at level l, $r^{\left(l\right)}$ is the output of the activity units denoted as the prediction, and $U^{\left(l\right)}$ specifies the matrix of synaptic weights that determine the prediction strength. The model treats $r^{\left(0\right)}$ as the direct sensory input and iterates through levels to minimize E.

Consequently, the predictions $r^{\left(l\right)}$ at each level l update over time according to


$$\begin{array}{c} \begin{array}{cc} & \frac{dr^{\left(l\right)}}{dt}=-\gamma \frac{\partial E}{\partial r^{\left(l\right)}}\\ & =-\gamma \left({\left(U^{\left(l\right)}\right)}^{T}e^{\left(l-1\right)}-e^{\left(l\right)}\right)\\ & =-\gamma \left({\left(U^{\left(l\right)}\right)}^{T}\left(r^{\left(l-1\right)}-U^{\left(l\right)}r^{\left(l\right)}\right)-\left(r^{\left(l\right)}-U^{\left(l+1\right)}r^{\left(l+1\right)}\right)\right). \end{array} \end{array}$$

Each level l updates its prediction, $r^{\left(l\right)}$, based on two factors: (1) the feed-forward input as the prediction error, $e^{\left(l-1\right)}$, from the preceding level, adjusted by the synaptic weight, $U^{\left(l\right)}$, represented by the solid orange line in Fig 3b, and (2) the difference between the current prediction, $r^{\left(l\right)}$, and the feedback from higher levels, $U^{\left(l+1\right)}r^{\left(l+1\right)}$, illustrated by the dotted blue line in Fig 3b. The update rate, γ, determines the extent to which these inputs influence the update of the current prediction, $r^{\left(l\right)}$, ensuring a balance between incorporating new data and preserving prior predictions. This equation methodically enhances predictions by applying both feedback and the propagation of errors and adjustments, highlighting the model’s adaptive learning capabilities.

The model updates the synaptic weights to reflect the learning process using gradient descent, expressed by


$$\frac{dU^{\left(l\right)}}{dt}=-\eta \frac{\partial L}{\partial U^{\left(l\right)}}=-\eta \left(e^{\left(l-1\right)}r^{\left(l\right)}\right),$$

where η represents the learning rate. This equation applies the Hebbian rule [29], demonstrating that the weight update is proportional to the product of the post-synaptic activity, $r^{\left(l\right)}$, and the pre-synaptic activity, $e^{\left(l-1\right)}$. The model iteratively adjusts the weights until it converges to an optimal set of weights, indicated by a minimum prediction error.

The current study employs a two-level classical predictive coding algorithm. The model updates the dynamics of the activity unit in predictive coding using


$$\frac{dr^{\left(1\right)}}{dt}=-\gamma \left({\left(U^{\left(1\right)}\right)}^{T}\left(x-U^{\left(1\right)}r^{\left(1\right)}\right)-\left(r^{\left(1\right)}-U^{\left(2\right)}r^{\left(2\right)}\right)\right),$$

$$\frac{dr^{\left(2\right)}}{dt}=-\gamma \left({\left(U^{\left(2\right)}\right)}^{T}\left(r^{\left(1\right)}-U^{\left(2\right)}r^{\left(2\right)}\right)\right)\;,$$

where $r^{\left(l\right)}$ represents the output of the activity unit, and x denotes the input data, specifically the positions of objects within the environment. The variable, $U^{\left(l\right)}$, specifies the synaptic weight that modulates the influence of activity units across the network levels, with subscripts 1 and 2 indicating the first and second level indices, respectively. This hierarchical update process integrates information across levels: the update of $r^{\left(1\right)}$ depends on the current estimates of $r^{\left(2\right)}$ along with the synaptic weights, $U^{\left(1\right)}$ and $U^{\left(2\right)}$. Similarly, the update of $r^{\left(2\right)}$ incorporates the current estimates of $r^{\left(1\right)}$, $U^{\left(1\right)}$, and $U^{\left(2\right)}$. This iterative interaction ensures dynamic adjustments within the network, enhancing predictive accuracy at both levels.

The model assumes that synaptic weight updates occur on a slower time scale than the rapid updates of neural activities. This assumption reflects the physiological understanding that synaptic plasticity modifies synaptic weights over extended periods and drives long-term learning processes. In contrast, neural activity involves neurons firing in response to stimuli and operates on a much shorter time scale, enabling rapid information processing on a trial-by-trial basis.

To draw a parallel with kinematics, consider the dynamic equation $x\left(t\right)=-\frac{1}{2}at^{2}+vt+x_{0}$, which relates an object’s position, x(t), over time, t, to its acceleration, a, and velocity, v, with $x_{0}$ as the initial position. Our model aligns $r^{\left(2\right)}$, representing an estimate of the system’s acceleration, with the acceleration term, a, and matches $r^{\left(1\right)}$, representing an estimate of velocity, to the velocity term, v. This interpretation emerges because lower-level representations, like $r^{\left(1\right)}$, respond to immediate changes in input data, similar to velocity, while higher-level representations like, $r^{\left(2\right)}$, capture slower, broader adjustments, akin to acceleration in kinematics.

The analogy highlights the hierarchical nature of the model, with $r^{\left(1\right)}$ (velocity) responding directly to input data changes and $r^{\left(2\right)}$ (acceleration) representing generalized, slower-changing features of dynamics. Our main assumption posits that the motion of each object results from the weighted sum of its motion sources, interpreting each motion source as representing velocities. The model leverages the predictive coding framework to represent each motion source, where $r^{\left(1\right)}$ in each predictive coding unit corresponds to the motion source, and the synaptic weight, $U^{\left(1\right)}$, quantifies the influence of each motion source on the object.

At each time step, the model forwards the estimated motion derived from the predictive coding framework to the surround suppression mechanism for refinement. The following section explains the surround suppression mechanism’s operation within the predictive coding framework.

### 2.4. Surround suppression mechanism

**Fig 4:**
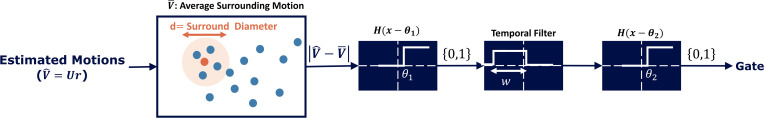
Surround Suppression mechanism. This diagram compares motion estimation at the center of the receptive field, denoted by $\hat{v}=Ur$, with the average motion in the surrounding area, denoted by $\bar{V}$, at each temporal step. This comparison uses a Heaviside function with the threshold parameter, $\theta_{1}$. The result is then integrated with the historical average motion within a temporal width window, w. This integrated value is again subjected to comparison, passing through a Heaviside function with a threshold parameter, $\theta_{2}$. The output is binary, indicating the activation state of the surround suppression mechanism.

The proposed model places surround suppression at its core, as illustrated in Fig 4. This mechanism modulates the response of motion-sensitive neurons to a stimulus within the central receptive field based on concurrent motion stimuli in the surrounding areas, aligning with principles outlined by [2].

The model links center-surround modulation to extra-classical receptive field effects observed in cortical neurons, particularly in the middle temporal (MT) area, rather than retinal ganglion cells (RGCs), where such interactions are more commonly associated. In cortical neurons, a stimulus in the surround does not directly elicit a response but instead modulates the response to the center stimulus. This modulation works antagonistically for direction-selective neurons: surround motion aligned with the center’s preferred direction suppresses activity while opposing motion enhances it [18]. The model further postulates that the surrounding suppression mechanism’s historical activity also influences this modulation.

The surround suppression mechanism stays inactive when the motion of an individual object aligns with the motion within the surround. The mechanism activates when a misalignment occurs between the surround and the object’s motion estimate. The computation incorporates weighted historical activity to account for temporal dynamics. When activated, the surround suppression mechanism suppresses the neuron’s activity. Conversely, when it remains inactive, the mechanism does not suppress the object’s estimate in the center of the receptive field, allowing the model to distinguish the object’s motion from the surrounding activity.

To implement the surround suppression activation function, the model compares the motion estimation of an object, $\hat{v}=Ur$, with the average motion observed within the object’s surroundings, which the model assumes to be circular with a diameter of d. The model defines this average motion as


$$\bar{v_{i}}=\frac{1}{Q}\sum_{o\in q_{i}}\sum_{j}u_{o,j}r_{j},$$

where $q_{i}$ defines the set of indices corresponding to objects within the circular area surrounding object i, and Q represents the total number of these surrounding objects. The double summation computes the combined influence of all motion sources (indexed by j) on each surrounding object, o, estimating their average motion in the surround.

In our model, the surround suppression mechanism accounts for motion within a specific region around the center of the receptive field. This method treats all surrounding objects as contributing equally to the calculation, regardless of their spatial distance from the receptive field center. While this equal-weight approach ensures computational efficiency, it simplifies the model by not accounting for distance-dependent synaptic variability. However, we did not enforce specific patterns on the shapes of synaptic activity based purely on distance. Instead, the synaptic weights are shaped through a learning process that reflects how neurons respond to motion over time, integrating the influence of surround motion in a biologically plausible manner. A distance-weighted averaging scheme in future studies could improve the model’s biological realism by accurately representing synaptic interactions.

The surround suppression module governs its behavior by actively integrating current and historical motion data through the function


$$f\left(\hat{v}\right)=H\left(H\left(\left|\hat{v}-\bar{v}\right|-\theta_{1}\right)-{\bar{f}}_{w}\left(\hat{v}\right)-\theta_{2}\right),$$

where the Heaviside step function, H(.), determines the activation state. The module evaluates the motion similarity by comparing the individual object’s motion, ${\hat{v}}_{i}$, with the average motion of its surrounding objects, ${\bar{v}}_{i}$, using the threshold, $\theta_{1}$. It further incorporates historical influence by applying the secondary threshold, $\theta_{2}$, which modulates the current response based on the historical average of suppression activation. The module uses these thresholds to actively assess and adjust the surround suppression mechanism, balancing immediate and historical factors to refine its output.

The parameter, $\theta_{1}$, plays a pivotal role by setting the threshold for surround suppression, determining how the model compares the motion within the center of the receptive field to the collective motion in the surrounding visual field. Increasing $\theta_{1}$ raises the threshold for surround suppression, requiring a larger difference between the estimated and average motion in the surround to activate the suppression mechanism. This adjustment directly influences how the model prioritizes or suppresses motion signals based on their divergence from the surrounding context.

The historical average of surround suppression activation, ${\bar{f}}_{w}$, is calculated over a specified time window, w. This temporal filter integrates information from past suppression activations, ensuring the model captures temporal dynamics in motion prediction. By broadening the window, w, the model incorporates a more extensive history of motion data, reducing the influence of transient noise and enhancing the stability of coherent motion detection. The ‘window duration’ refers to the size of the temporal window used in the temporal filter, as expressed in [eq:SD]. This window size determines how much past information from the surround suppression mechanism is considered during motion detection. The temporal filter integrates information over this window to allow the model to account for prior motion activity in the surrounding regions. Here, noise reflects variability in external sensory input, such as random dot motion, and its impact on simulated neuronal responses. The model excludes internal neural noise, such as variability in firing rates or synaptic activity, to simplify the dynamics and focus on the core processes being studied.

This study examines how the parameters, $\theta_{1}$ and w, affect the model’s ability to detect coherent motion. The model uses $\theta_{1}$ to process immediate motion within its receptive field and relies on w to define the duration of the temporal window for evaluation. While $\theta_{2}$ governs the integration of past motion information and influences how the model distinguishes relevant from irrelevant historical movements, this study does not focus directly on $\theta_{2}$. Instead, it indirectly addresses its effects through the temporal window duration, w. Our primary objective is to assess how incorporating historical motion data improves the model’s capacity to identify coherent motion in real-time sensory input. By fine-tuning the balance between immediate sensory cues and accumulated historical data, we aim to enhance the model’s robustness and accuracy in detecting coherent motion patterns.

## 3. Mechanistic framework of the model

The proposed model is grounded in a two-level predictive coding framework enhanced by surround suppression, designed to iteratively refine motion estimates. Predictive coding is the core computational strategy, integrating sensory inputs with prior knowledge to facilitate motion detection. Surround suppression introduces two key parameters—windows and thresholds—that shape this process. Inspired by neural mechanisms, these parameters reflect biological processes such as synaptic integration and inhibition. The windows of integration accumulate historical motion data over a temporal span, mimicking how neural circuits process inputs over time. Thresholds define the minimum motion contrast between the center and the surround required to activate suppression, capturing spatial and temporal modulation of neuronal excitability.

Temporal integration further enhances the model by incorporating the historical activation of surround suppression within a specific time window, creating a robust understanding of motion patterns over time. If suppression was previously activated during this period, it influences current neuronal activity, simulating the impact of prior information on motion detection. The model effectively simulates real-time motion detection while considering historical visual inputs by combining spatial integration through vector analysis with temporal integration through experience binding.

The hierarchical framework distinguishes between individual and shared motion. Individual motion arises from the first level of predictive coding, assessing the local motion of objects based on direct sensory input. In contrast, shared motion emerges after applying surround suppression, which integrates neighboring motion cues to identify global patterns. The model does more than simply reproduce the well-known fact that higher-level representations tend to become more global. The contribution of this model lies in the ability to simulate the intricate balance between local motion detection and global motion integration by incorporating both predictive coding and surround suppression. This nuanced approach allows for the decomposition of motion structure, as demonstrated in experiments such as the Random Dot Kinematogram (RDK), where shared motion emerges from noise-dominated environments, and the complex structure of the Johansson and Dunker wheels experiments. The model advances our understanding by quantitatively demonstrating how this interplay between local and global motion components evolves over time.

Surround suppression is crucial in identifying coherent motion patterns while suppressing noise or conflicting inputs. The model computes the prediction error at each stage—the discrepancy between the estimated motion and actual sensory input—which is propagated to the next stage for iterative refinement. The surround suppression mechanism specifically targets the detection of global motion in stimuli. By comparing motion signals between the center and the surrounding areas of the receptive field, the model activates suppression to extract shared motion in the visual scene. This spatial analysis, represented as vector comparisons, detects deviations or consistency in motion patterns, forming the foundation for identifying coherent motion signals.

The model incorporates synaptic weight modulation across two timescales to simulate neural adaptability. The short-term modulation captures immediate changes in synaptic strength in response to stimuli, although it is not as fast as the real-time changes in neural activity. This allows our model to reflect how synaptic weights can adjust over the course of a single trial. Additionally, we have incorporated long-term synaptic plasticity by relying on past information to adjust the synaptic weights over extended periods. This dual approach allows the model to simulate both rapid responses to new sensory input and the gradual influence of prior experiences on motion detection. We recognize that modeling the interactions among motion sources using synaptic weights is an abstraction and does not fully capture the distinct physical and temporal differences between these processes. The correspondence between synaptic weight changes and neural activity modulation in our model is intended to be instrumental, aiding in exploring noise’s role in motion detection and related deficiencies.

The hierarchical structure of the model reflects the brain’s ability to process global and local motion simultaneously. The initial module prioritizes global motion to establish a contextual foundation, while subsequent modules refine shared motion representations and resolve individual motion. This structure captures the dynamic interaction between global contextual processing and local precision. By abstracting motion interactions through synaptic weight changes, the model provides a functional framework for studying the impact of noise on motion detection and understanding hierarchical neural processing in dynamic environments. The combination of spatial and temporal integration ensures the model effectively simulates real-time motion detection while incorporating prior experiences.

### 3.1. Stimuli

We evaluated the algorithm using simulation results from three psychophysical experiments: (I) the Johansson experiment [9], (II) the Duncker wheel [21], and (III) the Random Dot Kinematogram [30]. Each experiment contributes uniquely to the study of motion perception and processing.

**Fig 5:**
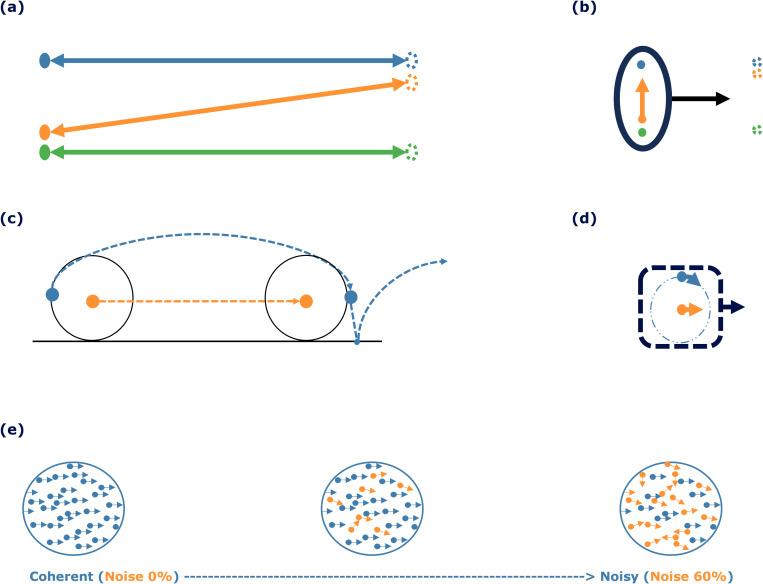
Overview of experimental paradigms. (a-b) A schematic view of Johansson’s experiment with three dots, showing (a) motion vectors for each of the dots and (b) the perceived motion of the group (denoted by the oval containing the three dots moving rightwards) and the central dot (moving upwards) [14]. (c-d) A schematic view of Duncker wheel’s experiment with three dots showing (c) motion vectors for each of the hub- and rim-dots and (d) the perceived motion of the group (denoted by the dashed square containing both dots moving rightwards) and the rim-dot (moving in a circular motion) [14]. (e) Random Dot Kinematogram. The difficulty of the experiment escalates from left to right with an increasing number of noise dots, depicted in orange (although the dot colors are not different in the experiment). The blue dots represent signal dots that have coherent motion towards the right.

In the Johansson experiment (experiment I) [9], illustrated in Fig 5a and 5b, three vertically aligned dots move back and forth across a display. The two outer dots move horizontally, while the middle dot moves diagonally, maintaining collinearity with the outer dots (Fig 5a). Observers perceive the middle dot as moving vertically, oscillating between the two horizontally moving outer dots, despite its actual diagonal trajectory (Fig 5b). This illusion arises from the synchronized horizontal motion of all three dots, where the brain interprets movement based on their relative positions and synchronized movements.

The Duncker wheel experiment (experiment II) [21] explores motion perception by featuring two dots placed on an invisible wheel, as shown in Fig 5c and 5d. One dot, positioned at the hub of the wheel, moves linearly (typically left to right), while the other dot follows a complex spiral path along the rim due to the wheel’s rotational and lateral movement (Fig 5c). Observers perceive these motions differently: they see the hub dot moving linearly, consistent with its actual motion, but perceive the rim dot, despite its spiral path, as moving in a simple circular motion around the hub dot (Fig 5d). This phenomenon demonstrates the brain’s tendency to break down complex movements into recognizable components, interpreting the spiral motion of the rim dot as a combination of translation and rotation when the hub dot serves as a reference point.

The Random Dot Kinematogram (RDK) (experiment III) serves as a valuable method for investigating motion perception [30]. In the RDK, numerous small dots move randomly across a screen, as shown in Fig 5e. Among these, a subset of dots (blue) moves coherently in the same direction, while the remaining dots (orange) move randomly. Observers are tasked with identifying the direction of coherent motion. For example, in a display with 95% coherence, 95% of the dots (signal dots) move in a designated direction between frames, while the remaining 5% (noise dots) move randomly. Higher coherence levels facilitate the perception of global motion direction. This experimental design provides critical insights into how the visual system processes and differentiates motion information from noise. The concept of ‘time to convergence’ is introduced to evaluate model performance in this context. This refers to the number of discrete time steps, each with Δt duration, required for the model to identify the direction of coherent motion within the RDK accurately. Coherent motion is considered accurately identified when the motion strength stabilizes, with variations remaining below 0.05 units across successive time steps. This ensures the model converges to a stable and reliable representation of coherent motion direction.

The computational time (a surrogate measure of latencies) measured in the model does not correspond directly to the time required for cognitive processing in the brain. Instead, the model provides an abstraction of the underlying mechanisms involved in motion detection rather than replicating the exact timing of neural responses. In biological systems, multiple brain regions, including attention mechanisms and higher-order decision-making, contribute to motion perception and influence response times. Accurately modeling these temporal dynamics would require a more detailed framework that includes the complexities of cortical interactions, synaptic plasticity, and attentional modulation, which lies outside the scope of this work.

The parameters of the stimuli used in the experiments are summarized in Table 1. The subsequent section presents the results of the model simulations and their role in elucidating key aspects of human visual motion perception as explored through these psychophysical experiments.

**Table 1:** Parameters of stimuli used in the study.

Stimulus	Parameter	Value
	Frequency	0.5 Hz
Johansson Experiment	Horizontal Amplitude	$2\sqrt{0.3}$
	Vertical Amplitude	$cos\left(45^{\circ }\right)2\sqrt{0.3}$
	Time Constant	1/60 s
	Wheel Radius (R)	10 unit
Duncker Wheel	Horizontal Speed	0.5 unit/s
	Angular Speed	0.1 deg/s
	Time Constant	0.05 s
	Number of Dots	1000
	Dot Density	500 dot/unit
Random Dot Kinematogram	Coherent Velocity	0.01 unit/s (horizontal motion)
	Random Velocity	0.01 unit/s (vertical motion)
	Time Constant	0.05 s

## 4. Results

We evaluated the model’s performance in identifying motion structures and extracting coherent motion across three distinct experiments. Furthermore, we analyzed how prior knowledge affects the model’s performance with motion data and examined the role of the surround suppression mechanism in detecting coherent motion.

### 4.1. The model successfully identified the motion structure in classical motion patterns

**Fig 6:**
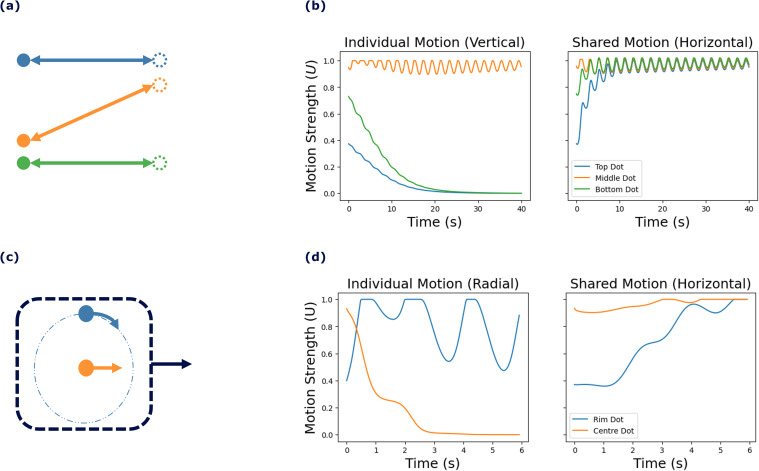
The model successfully identified the motion structure in experiments I and II. (a) A schematic view of Johansson’s experiment I [9] with three dots. (b) Model estimates of motion strength: shared and individual, horizontal and vertical motions, respectively. The individual motion strengths reach zero for the two horizontal moving dots (blue and green), but is close to maximum for the middle dot (orange). (c) The Duncker wheel experiment II [21] with a central dot (orange) and a dot on a circle (blue). (d) The model recognizes shared motion (horizontal motion) and an individual element (rotational motion) of the rotating dot. The plots are color coded, matching the colors of the objects with their corresponding plot lines.

Fig 6 illustrates the results of classical motion perception experiments simulated using our predictive coding framework. The model successfully captured the motion structures in the Johansson [9] and Duncker [21] experiments (I and II), effectively distinguishing between shared and individual motion types.

Psychophysical experiments on Johansson motion in experiment I (see Fig 6a) show that humans perceive this stimulus as shared horizontal motion, with the central dot oscillating vertically [9]. Based on these observations, we expect the model to extract equal (synchronous) shared motion across all three dots and to identify individual motion specifically for the central dot. The model’s output consists of connectivity weights U, which represent the strength of the contribution of each motion source (motion strength). Fig 6b displays the estimated motion strengths for shared and individual motions. The individual motions of the blue and green dots converge to zero over time, while the individual motion of the central dot (orange) remains pronounced. The shared motion of all three objects converges to similar values, close to 1. These results demonstrate the model’s ability to distinguish between shared motion, where all dots synchronize, and individual motion, which diminishes for the outer dots but persists for the central dot. The observed oscillation in motion strength arises from the sinusoidal motion of the objects as they move back and forth horizontally. In this experiment, shared motion corresponds to the collective horizontal movement of all dots, causing synchronized oscillation. Individual motion is characterized by vertical movement, which is absent for the blue and green dots (vertical motion strength is zero) but present for the orange dot, as indicated by its non-zero motion strength.

The application of the model to the Duncker experiment in Experiment II (Fig 6c) validates its ability to represent complex motion patterns. As illustrated in Fig 6d, the model successfully captured the shared horizontal motion of the dots while distinguishing the individual motion of the rim dot (blue) through its significant individual motion strength. In contrast, the other horizontally moving dot (orange) exhibited an individual motion strength that converged to zero, effectively simulating the perception of a rolling wheel. In this experiment, shared motion corresponds to the horizontal movement of both dots, causing them to move horizontally to the left. Individual motion, interpreted as radial motion, converges to zero for the horizontally moving dot but remains non-zero for the rim dot (blue). The pronounced oscillation observed in the shared motion of the radial ball, resulting from the consistent value of the motion source, effectively represents the motion strength of the radial movements of the blue dot. The period and amplitude of the sinusoidal motion strength output vary with several experimental variables. For example, changes in the positions of dots and the duration of their movement directly influence the amplitude and frequency of the oscillation. When the objects follow a more structured pattern or move over a longer duration, the model produces a more stable oscillation with a higher amplitude, accurately reflecting the motion’s dynamics.

**Fig 7:**
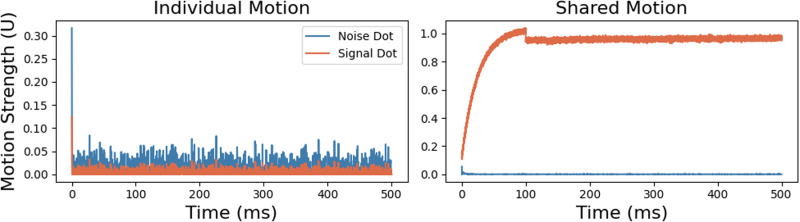
Individual and shared motion in a random dot kinematogram, experiment III. Blue lines represent the motion strengths of noise dots, while the orange lines depict the motion strengths of signal dots with coherent motion. Note the reduced scale axis of the individual motion plot, used to highlight the low motion strengths of the dots.

Fig 7 presents the results from a Random Dot Kinematogram (RDK) in experiment III, which illustrates the distinction between individual and shared motion. Noise dots (dots with noncoherent motion, blue line) exhibited higher and more variable individual motion strengths compared to signal dots (orange line). Despite this, the large number of noise dots prevented their individual motion strength from reaching 1. The mean individual motion strength for noise dots (10.4 units/ms) exceeded that of signal dots (3.25 units/ms), with noise dots displaying significant fluctuations while signal dots maintained a relatively stable, lower value. This contrast underscores the unpredictable and incoherent movements of noise dots compared to the coherent motion of signal dots. In the shared motion graph, signal dots quickly reached and sustained a high shared motion strength close to 1, whereas noise dots exhibited almost no shared motion. This indicates that the model successfully isolated the coherent motion from the random background. As the model stabilized, the individual motion strengths of signal dots diminished to zero, demonstrating the successful detection of coherent motion. The drop in motion strength for the signal dots at approximately 100 ms likely marked the point at which the model begins to recognize not only the shared motion but also the individual noisy motion of the dots. Up to 100 ms, the model primarily reinforced the presence of shared motion in the stimuli. Beyond this point, the model incorporated the contribution of individual dot motion, explaining the observed decline in overall motion strength. This dynamic highlights the model’s ability to adjust and integrate motion information over time.

### 4.2. The balance between sensory data and prior knowledge in coherent motion detection

**Fig 8:**
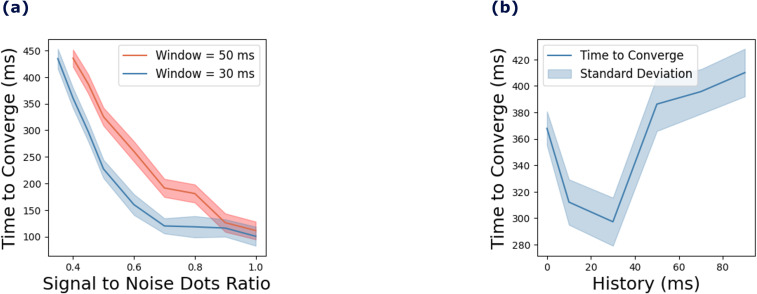
Effects of noise and historical window duration on the time to convergence of model (experiment III). (a) The time to converge as a function of signal to noise dots ratio for two different historical window durations: 50 ms and 30 ms. (b) Convergence time dependence on the historical window duration. The shaded areas around the lines represent the standard deviations across 150 trials for each window duration.

The model’s performance in detecting coherent motion in the RDK task was assessed by measuring the time to convergence. Fig 8 illustrates variations in convergence time as noise levels in the stimuli change, along with the impact of prior knowledge on the model’s performance. Fig 8a shows a strong relationship between the signal-to-noise dots ratio and convergence time, with higher noise levels leading to longer convergence durations. This experiment was conducted for historical window durations of 0, 10, 30, 50, 70, and 90 ms, with 150 trials per window duration.

Fig 8a compares the effect of signal-to-noise dots ratio on convergence time for two historical window durations: 50 ms and 30 ms. The longer historical window resulted in longer convergence times compared to the shorter window. The shaded regions in the figure indicate the standard deviation across the 150 trials.

Fig 8b depicts the relationship between historical window duration and convergence time for a signal-to-noise dots ratio of 0.4. Convergence time initially decreased as the historical window duration increased, reaching an optimal point at 30 ms. Beyond this point, further increases in historical window duration led to a rise in convergence time. These findings suggest an optimal range of historical information that enhances the detection of coherent motion. Exceeding this range appears to hinder the model’s performance, likely due to an over-reliance on historical data.

We presented results for window durations of 30 ms and 50 ms, as these durations demonstrate the model’s performance across varying noise proportions. We chose to highlight the 30 ms window because it achieves an effective balance between integrating past information and maintaining a computationally efficient time to convergence. As shown in Fig 8b, the time to converge increases with higher noise proportions, yet the model exhibits a consistent trend across different noise levels. The results for the 30 ms and 50 ms windows demonstrate stability under varying noise conditions, indicating the robustness of the model. These findings suggest that the model effectively adapts to different noise levels while maintaining reliable performance across these historical window durations.

### 4.3. Surround suppression and the detection of coherent motion

The model’s performance was assessed by modifying the influence of surround suppression on the detection of coherent motion in the RDK task (experiment III). Specifically, we investigated the effect of adjusting the threshold parameter, $\theta_{1}$, in the surround suppression mechanism. A higher threshold value indicates that the motion in the center and surround must differ by more for the surround suppression mechanism to activate.

**Fig 9:**
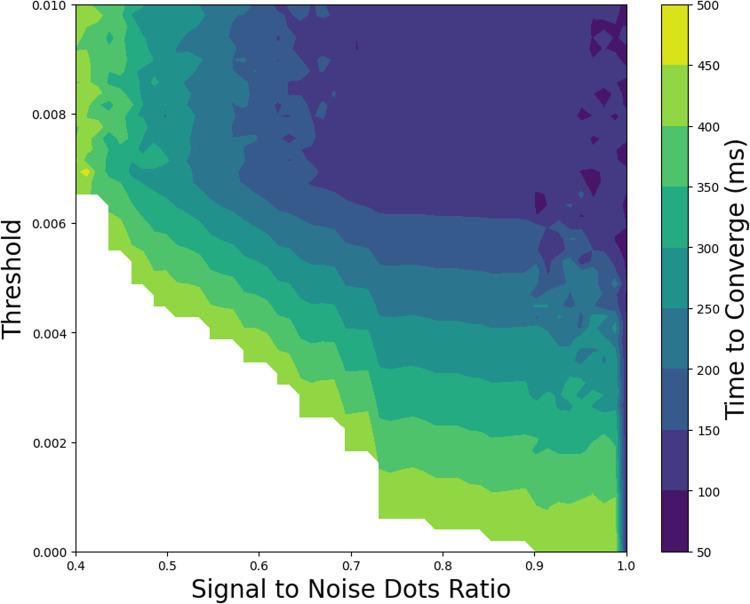
Effects of noise level and surround suppression threshold on model convergence time in the RDK task, experiment III. The color indicates the convergence time of the model in ms, with cooler colors representing shorter times and warmer colors indicating longer times.

Fig 9 summarizes the model’s performance in the RDK task. The axes represent the surround suppression threshold and the signal-to-noise dots ratio. The color gradient reflects the convergence time, with cooler colors indicating shorter durations and warmer colors indicating longer durations. The figure demonstrates that, as noise levels increase, the time to converge also increases, indicating that the model requires more time to identify coherent motion directions under higher noise conditions.

The results also highlight the impact of the surround suppression threshold on convergence time. Lower thresholds result in longer convergence times, while increasing the threshold to sufficiently high levels enhances the model’s performance in noisy environments. Higher thresholds reduce the impact of noise on the surround suppression mechanism, preventing excessive changes due to random noise and improving the model’s ability to process coherent motion.

## 5. Discussion

This study presents a biologically plausible computational model based on the predictive coding algorithm for coherent motion detection. The model extracts motion structure from the visual field by decomposing motion into individual and shared sources, providing insights into coherent motion perception. It integrates core processes such as predictive coding and surround suppression, fundamental mechanisms that link neural processes to behavioral outcomes. Although the model adopts an abstract approach without explicitly simulating specific neurons or cortical layers, it offers a broad and adaptable framework for understanding motion detection.

### 5.1. Decomposing the motion structure into shared and individual motion sources

Testing the model with various stimuli highlights the significance of incorporating a surround suppression mechanism into the motion detection process. Classical predictive coding provides some degree of surround suppression, but it fails to capture fully the key characteristics of extra-classical receptive field effects necessary for accurately interpreting the complex interplay between object motion and its neighbors. Neurophysiological studies show integrative and antagonistic effects, where motion in the surround influences neuronal activity [3,31,32]. In the antagonistic case, neurons increase their activity when motion in the center and the surrounding area occur in opposite directions. Conversely, in the integrative case, neurons suppress their activity under similar conditions [2,3,6,19,32,33]. Our method incorporates this integrative effect to offer a more nuanced and comprehensive model for analyzing motion interactions within dynamic scenes. Including this mechanism is crucial for decomposing the motion structure and extracting shared motion within the visual field.

Center-surround interactions in the middle temporal (MT) area are critical for motion perception, as the activity of direction-selective neurons is modulated by stimulus alignment with its surround through suppression or enhancement [4–6,34,35]. These mechanisms evaluates motion in both the center and the surround of the receptive field to check for consistency in the visual field. It suppresses neuronal activity when motion in the center and the surround occur in different directions [2,19,31]. This contributes significantly to coherent motion perception [18]. Studies demonstrate that the center-surround mechanism plays a pivotal role in figure-ground segregation, edge detection, and distinguishing the movement of two overlapping or nearby objects within the receptive field [3,6,32,33]. This model underscores the importance of such mechanisms for detecting global (shared) motion in the visual field. Implementing the center-surround mechanism in the model, based on the predictive coding algorithm, demonstrates this critical function effectively. Increasing the threshold for surround suppression improves the model’s performance in detecting coherent motion. Conversely, decreasing the threshold for surround suppression suppresses neuronal activity and increases the model’s convergence time.

We tested the model using three distinct types of stimuli known for their significant contributions to the study of global motion detection in the visual cortex: (I) the Johansson experiment, (II) Duncker wheel, and (III) random dot kinematogram. The Johansson experiment I shows how the brain integrates information from multiple points into a coherent perception of global motion. The Duncker wheel experiment II demonstrates the tendency of our brain to break down multiple complex movements into recognizable, coherent motions, such as that of a rotating wheel in this experiment. The random dot kinematogram experiment III allows motion coherence to be manipulated by changing the level of noise to study how the brain integrates or segregates motion signals from different spatial locations. The proposed model effectively decomposes motions within the visual field into shared and individual object motions across all these stimuli, replicating the human brain’s perception of global motion (see Fig 6b and 6d). By implementing a predictive coding algorithm, the model illustrates how the brain predicts an object’s future position by using its current and past states of motion, aligning with kinematic principles.

This study underscores the application of predictive coding in explaining motion perception while laying the groundwork for future research. Expanding the model to incorporate internal noise dynamics, distinctions among cortical areas, and direct links between neural mechanisms and cognitive functions could provide deeper insights into typical and atypical motion perception. By bridging these gaps, this work offers a compelling framework for advancing the understanding of motion detection and its related impairments.

### 5.2. Balancing sensory data and prior knowledge in the detection of coherent motion

The modeling results demonstrate that increasing noise levels in the random dot kinematogram experiment III delays the model’s convergence time for detecting the global motions of the dots (see Fig 8a). This finding replicates the psychophysics experiment by Spencer et al. [36], which showed that participants need longer duration stimuli to detect global motion at higher noise levels. Their experiment compared healthy controls with patients with schizophrenia in detecting coherent motion in random dots. They found that patients with schizophrenia required longer durations than healthy participants to detect global motion across varying noise levels. The model indicates that increasing the influence of prior knowledge during motion detection significantly affects the convergence time. Extending the duration of the window of past information in motion detection further prolonged the model’s convergence time, as shown in Fig 8a.

We analyzed this phenomenon further by varying the window’s duration for utilizing past information from 0 to 100 ms. The results revealed that increasing the window duration to a minimum threshold of 30 ms optimizes the model’s performance and reduces the time to convergence. Extending the window duration beyond this 30 ms threshold gradually lengthens the convergence time and ultimately causes the model to fail to converge, as illustrated in Fig 8b. This finding highlights the importance of balancing prior knowledge and incoming sensory data in detecting coherent motion. The results demonstrate that the model must avoid overly relying on past information (i.e., a very large window duration) to detect coherent motion effectively. For optimal performance, the model requires some past information to prevent it from responding excessively to noise. Reducing the influence of past information (i.e., a very small window duration) increases the model’s convergence time, as the noise in the stimuli overly disturbs its detection of coherent motion.

### 5.3. Comparisons with existing computational models of motion perception

The model developed by Bill et al. [7] and Gershman et al. [14] offers valuable insights into the hierarchical inference of visual motion structure by using a Bayesian framework to represent the relationships between multiple motion components. Their approach integrates sensory inputs with high-level probabilistic inference to explain how the brain extracts coherent motion from complex visual stimuli. Our model, grounded in the predictive coding framework, pursues the same objective of representing hierarchical motion perception. However, our model explicitly incorporates a surround suppression mechanism inspired by the neurobiological process of motion integration observed in MT cells. This process modulates motion signals based on their contextual relationship with surrounding stimuli [2,6,18]. This mechanism plays a critical role in distinguishing between shared and individual motions in dynamic environments, an aspect not explicitly addressed in the model by Bill et al. [7] and Gershman et al. [14]. Furthermore, while their model captures motion structure through Bayesian inference, our model also leverages the balance between prior knowledge and incoming sensory data to replicate motion detection processes.

Classical predictive coding models, such as that proposed by Rao and Ballard [16], explain several visual cortical responses, including extra-classical receptive-field effects. However, they do not fully address motion perception, which introduces additional complexities. Although Rao and Ballard’s model includes feedback for spatial prediction, it does not account for the dynamic aspects of motion stimuli or the role of surround suppression in motion detection. Our model addresses these limitations by extracting motion structure and accounting for interactions between center and surround motion signals, which are critical for accurate motion detection. Our model provides insights into how motion perception under noisy conditions requires mechanisms that integrate motion consistency and surround suppression to filter irrelevant motion signals. Unlike classical models, our approach emphasizes the temporal dynamics of motion and the importance of context-dependent modulation, particularly in scenarios where global motion is disrupted.

Recent models, like the dynamic predictive coding (DPC) framework introduced by [37], enhance our understanding of spatiotemporal prediction in the neocortex. Their model organizes sequence learning hierarchically, where neural processes in lower-level cortical structures predict short-term dynamics in higher-level cortical structures and higher levels generate abstract representations across longer timescales. This multi-level approach captures predictive and postdictive effects, offering insights into phenomena like the flash-lag effect [38]. While the DPC framework explains general hierarchical sequence processing and episodic memory formation, our model integrates surround suppression and predictive coding to simulate motion perception impairments under pathological conditions, such as schizophrenia. Our model emphasizes how alterations in motion signal integration, particularly through surround suppression, contribute to deficits observed in patients with schizophrenia. By focusing on clinical phenomena like increased latency in motion detection and over-reliance on past information, our model complements the DPC framework while providing a specialized focus on perceptual abnormalities in mental disorders. No previous model has explicitly showed this connection, making our contribution both novel and significant. Furthermore, this understanding could pave the way for developing clinical or diagnostic tools to better assess and manage motion perception impairments in schizophrenia, potentially aiding in early detection and personalized interventions.

### 5.4. Relationship between model results and motion perception in schizophrenia

Our model successfully emulates key findings from psychophysical experiments, particularly in extracting motion structures observed in the seminal studies by [9] and [21]. Despite significant advancements in understanding motion perception, few studies have explored this domain in the context of schizophrenia, leaving a critical gap in the literature. Our simulation aligns with the observed differences in stimulus duration required by patients with schizophrenia compared to healthy controls for detecting coherent motion. Prior research has shown that individuals with schizophrenia often require more prolonged exposure to detect global motion in noisy environments compared to healthy controls [36]. By adjusting the influence of prior knowledge and the strength of surround suppression, our model provides a novel explanation for these deficits, offering valuable insights into the neural basis of motion perception differences between healthy individuals and those with schizophrenia.

These results suggest a possible explanation for the motion detection deficiency observed in patients with schizophrenia. Studies show that distortions in perception and beliefs among individuals with schizophrenia arise from accurately encoding prior knowledge, which makes them less responsive to feedback (i.e., under-learning) [39–41]. Other theories propose that abnormalities in motion perception result from interpreting prediction errors as significant changes, leading to exaggerated responses to noisy information and rapid adaptation to a changing environment compared to controls (i.e., over-learning) [42,43]. A recent study reveals the coexistence of these conflicting theories, showing that the learning process in these patients is not stationary and depends on the statistical context. The applied task demonstrated both over-learning and under-learning in different situations [41]. The model developed here indicates that the motion detection deficiency in patients with schizophrenia may arise from the same mechanism, either by overly relying on past information (under-learning) or by excessively updating based on feedback (over-learning). This hypothesis aligns with broader theories suggesting that schizophrenia involves disruptions in balancing sensory input with prior expectations ([39,42,43];[40,41]). By incorporating these dynamics, the model provides a mechanistic explanation for the behavioral deficits observed in motion perception tasks.

**Fig 10:**
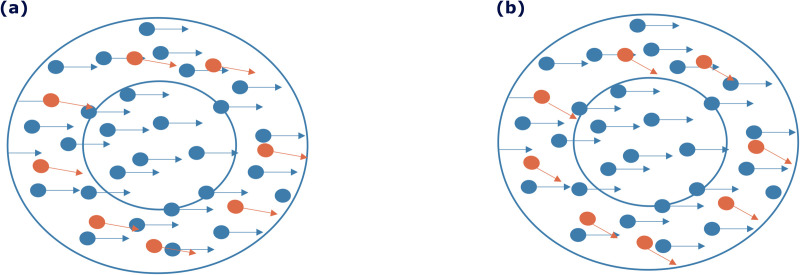
Stimuli to test the coherency threshold for surround suppression. (a) Two sets of overlapping random dots in the center (only blue dots) and the surround (blue and orange dots). Both sets of dots have coherent directions, except that the angle of the motion direction for the red dots is slightly different. (b) The same dots, but the angle of the difference between the motion directions for the red and blue dots is larger.

The model shows the effect of weaker surround suppression on motion perception and, for the first time, offers a mechanistic explanation for how motion detection deficiencies observed in some motion detection tasks might arise from this impaired surround suppression. This finding is crucial as it suggests a new hypothesis that observed weak surround suppression could underlie the motion detection deficiencies observed in patients with schizophrenia [44]. To further explore these deficits, we propose an experiment (illustrated in Fig 10) that investigates the effects of discordant motion in the surround on coherent motion detection. Participants would judge the direction of coherent motion in the center of stimuli, while the angle of difference between the motion of surround elements is systematically varied. Measuring the minimum duration required to detect coherent motion in the center would allow for a comparison between neurotypical individuals and patients with schizophrenia. This experiment could reveal how angular differences in surround motion impact motion detection and provide insights into the neural circuitry underlying surround suppression deficits. It is important to note that the current model is limited to simulating impairments specific to the MT area. Future work could extend this framework to incorporate features of upstream and downstream cortical structures to better understand the hierarchical processing deficits in schizophrenia.

Various computational models address schizophrenia by modeling neural circuitry, synaptic dysfunctions, and perceptual anomalies [45]. However, most models overlook specific motion detection deficiencies in patients with schizophrenia. Existing models typically concentrate on higher-order cognitive deficits, reward-based learning, and hallucination-like symptoms, with few addressing sensory-level impairments like motion perception. Our model fills this gap by recovering biophysical results for impaired motion perception comparable to those so far reported in MT cell functions for schizophrenia patients. This focus on low-level sensory processing anomalies represents an unexplored domain in computational psychiatry, complementing existing models of higher-order cognitive dysfunctions in schizophrenia research.

To our knowledge, no existing model specifically addresses motion detection deficiencies in schizophrenia within the predictive coding framework. While previous studies link predictive coding impairments to broader aspects of schizophrenia, such as cognitive and perceptual disruptions [26], our study is the first to focus on how these impairments manifest in motion detection.

## Supporting information

S1 Fig Effect of dot density on convergence time in coherent motion detection. The figure shows how increasing dot density affects convergence time.
(PDF)
