# Supplementary material for: Balancing prior knowledge and sensory data in a predictive coding model of coherent motion detection
Source: PLoS Comput Biol. 2025 May 21;21(5):e1013116. doi: 10.1371/journal.pcbi.1013116 (PMC12133192; doi:10.1371/journal.pcbi.1013116)
Supplement: S1 Fig — The figure shows how increasing dot density affects convergence time. (PDF) [file pcbi.1013116.s001.pdf]

Supporting Information

We investigated the effect of the number of dots on the model's computation and convergence time, Fig. The results show that while the number of dots impacts convergence time, the effect diminishes as dot density increases. Convergence time decreases and eventually stabilizes. This supports our hypothesis that the model's performance is robust due to its reliance on predictive coding and surround suppression mechanisms rather than the absolute number of dots.

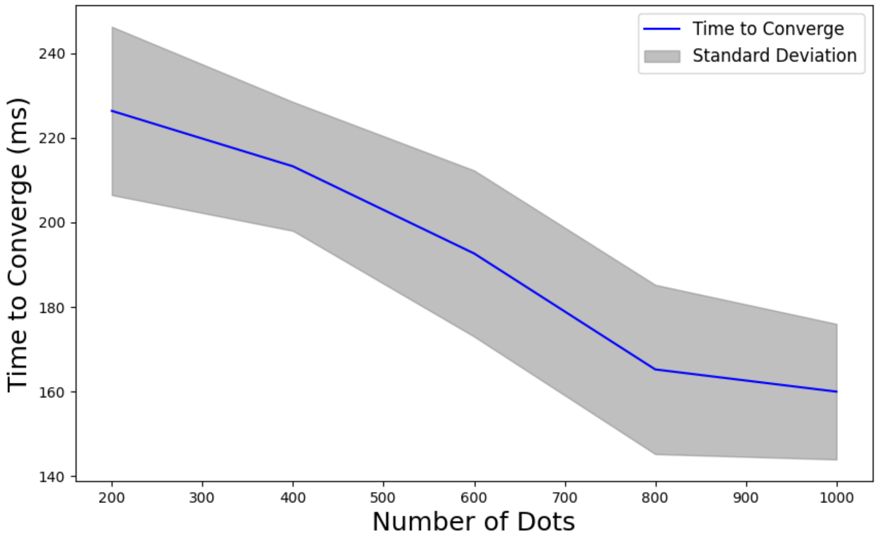

S1 Fig : **Effect of Dot Density on Convergence Time in Coherent Motion Detection.** The blue line represents the mean convergence time, while the shaded gray region indicates the standard deviation.
